# Inclusion of genetic variants in an ensemble of gradient boosting decision trees does not improve the prediction of citalopram treatment response

**DOI:** 10.1038/s41598-021-83338-2

**Published:** 2021-02-12

**Authors:** Jason Shumake, Travis T. Mallard, John E. McGeary, Christopher G. Beevers

**Affiliations:** 1 Department of Psychology, Institute for Mental Health Research, University of Texas At Austin, 305 E. 23rd St., E9000, Austin, TX 78712 USA; 2 Providence Veterans Affairs Hospital and Brown University School of Medicine, Providence, RI USA

**Keywords:** Psychology, Predictive markers

## Abstract

Identifying in advance who is unlikely to respond to a specific antidepressant treatment is crucial to precision medicine efforts. The current work leverages genome-wide genetic variation and machine learning to predict response to the antidepressant citalopram using data from the Sequenced Treatment Alternatives to Relieve Depression (STAR*D) trial (*n* = 1257 with both valid genomic and outcome data). A confirmatory approach selected 11 SNPs previously reported to predict response to escitalopram in a sample different from the current study. A novel exploratory approach selected SNPs from across the genome using nested cross-validation with elastic net logistic regression with a predominantly lasso penalty (alpha = 0.99). SNPs from each approach were combined with baseline clinical predictors and treatment response outcomes were predicted using a stacked ensemble of gradient boosting decision trees. Using pre-treatment clinical and symptom predictors only, out-of-fold prediction of a novel treatment response definition based on STAR*D treatment guidelines was acceptable, AUC = .659, 95% CI [0.629, 0.689]. The inclusion of SNPs using confirmatory or exploratory selection methods did not improve the out-of-fold prediction of treatment response (AUCs were .662, 95% CI [0.632, 0.692] and .655, 95% CI [0.625, 0.685], respectively). A similar pattern of results were observed for the secondary outcomes of the presence or absence of distressing side effects regardless of treatment response and achieving remission or satisfactory partial response, assuming medication tolerance. In the current study, incorporating SNP variation into prognostic models did not enhance the prediction of citalopram response in the STAR*D sample.

## Introduction

There is substantial variability in response to depression treatment. On average, 30–40% remit following initial treatment, although over time remission rates can climb as high as 70% with additional forms of treatment^1^. An efficient health care system would ideally deliver the most effective treatments as soon as possible and minimize trial and error. Thus, developing data-driven guidance about which treatments are most likely to work for patients with specific attributes is a high priority^2^.

Response to depression treatment is likely complex and multifactorial^3^. It is often posited that genetic variation may be an important individual difference that could predict response to depression treatment. Indeed, prior studies suggest that response to depression treatment is heritable. For instance, variation in common single nucleotide polymorphisms (SNPs) are estimated to explain 42% (*SE* = 0.180) of the variance in citalopram response^4^. This effect is likely highly polygenic, as chromosome length was associated with proportion of variance attributable to the chromosome. Moreover, other work has demonstrated that the heritability of citalopram response was not attributable to genetic variants commonly associated with serotonergic and dopaminergic signaling^5^.

Although treatment response appears to be heritable, this does not necessarily indicate that genetic variation will be a useful predictor of treatment response. While heritability determines associations between genetic variation and phenotypes at a population level, prediction relies on individual differences in genetic variation to predict treatment response. Indeed, attempts to identify specific genetic biomarkers of antidepressant treatment response, particularly when using candidate gene approaches, have had decidedly mixed results^6,7^.

Traditional genome-wide methods have been used to identify specific SNPs associated with depression symptom response following treatment with citalopram in the STAR*D trial^8^. Although no SNP reached genome-wide threshold for statistical significance, a recent study reported that three SNPs (rs6966038, rs6127921, rs809736) approached significance (*p* values less than 1 × 10^–5^). Thirty-nine additional SNPs had *p* values < 0.0001^9^. Other work identified eleven SNPs and six clinical variables in a training dataset that also predicted depression remission in response to escitalopram in an independent validation dataset with an area under the curve of 0.77 (95% CI; 0.66–0.88)^10^. Further, a polygenic score used to estimate antidepressant treatment response in one trial (GENDEP) did not predict response to antidepressant treatment response in a second trial (STAR*D) and vice versa^11^. Thus, despite the apparent heritability of antidepressant treatment response, the usefulness of SNP variation for the prediction of antidepressant treatment response remains unclear.

The current study examined whether the inclusion of SNPs in a machine learning stacked ensemble could improve the prediction of treatment response above and beyond the contribution of more standard clinical predictors in the STAR*D trial. Indeed, it has been speculated that the inclusion of genetic or brain-based predictors could further enhance the prediction of treatment response in this dataset^12^.

Building on prior work, we used two different approaches to select genetic predictors of treatment response. First, we selected the same SNPs (or proxy SNPs if the identical SNPs were unavailable) previously shown to predict response to escitalopram (the S-enantiomer of citalopram) in a different clinical trial^10^. (Given that the antidepressant effect of citalopram is due to the S-enantiomer^13,14^, predictors of response to escitalopram should be highly relevant to citalopram response as well.)

Second, we used a nested cross-validation approach with elastic net logistic regression to identify the most promising SNPs from across the genome to be used in the prediction models. No prior studies using data with the STAR*D trial have used this approach to select genetic variants for use in multivariate prediction models of treatment response.

For each approach to SNP selection we utilized between-clinic cross-validation (i.e., used data from 13 clinic sites to predict outcome in the 14th site and then repeated for each site) to provide a rigorous test of model generalizability. This allows for the examination of how well a prediction model derived from all but one clinical site generalizes to an unseen (i.e., out-of-fold) clinical site. This is akin to standard k-fold cross-validation, only the folds are determined by clinical site rather than random selection. For each approach to SNP selection, we examined whether the inclusion of the genetic variants improved the prediction of treatment response beyond model performance achieved by using pretreatment clinical and sociodemographic predictors only.

## Methods

### Participants

The current study involved participant data from the publically available STAR*D study^15,16^. Participants in the STAR*D trial met DSM-IV criteria for nonpsychotic major depressive disorder at study entry, were 18–75 years of age, not pregnant, not breastfeeding, and had not previously received any protocol treatment within the first two treatment steps of the study. Exclusion criteria included participants diagnosed with active suicidal ideation or substance use that required acute hospitalization, a primary diagnosis of bipolar, psychotic obsessive–compulsive and/or eating disorders, those with general medical conditions that precluded protocol medications and those who had shown nonresponse or intolerance to protocol medications within the current depressive episode prior to study enrollment. The current study used the Level 1 data from STAR*D. Written informed consent was obtained by STAR*D investigators from all participants during the STAR*D trial. In the current analysis of the publically available STAR*D data, all methods were carried out in accordance with relevant guidelines and regulations and approved by the Internal Review Board at the University of Texas at Austin.

Of the 4041 participants initially enrolled in STAR*D, 1948 provided DNA samples for genotyping. There were slight differences between the STAR*D participants who provided DNA samples and participants who did not. Genotyped participants were older, better educated, had higher household incomes and were more likely to be retired or married; however, depression scores did not significantly differ between groups [for more detail, see^17^].

Analyses were completed with the full sample who provided DNA with valid outcome data (n = 1663; *n* = 285 with missing outcome data) and sensitivity analyses were conducted on a sub-sample limited to participants of European-ancestry (identified via genomic principal component analyses; please see supplementary materials for more detail) (*n* = 1127). When SNPs were combined with clinical predictors, we further limited the sample to patients who received their baseline clinical assessment prior to starting citalopram (all ancestries *N* = 1257 and European-ancestry *N* = 827; *n*s = 406 and 300, respectively, who received their baseline clinical assessment after starting citalopram) because there is evidence that early clinical improvement predicts the likelihood of ultimate treatment response (The STAR*D trial also allowed patients to enroll if they were already taking citalopram, provided they had started no more than 2 weeks prior to enrollment.). Thus, limiting the sample to those who were assessed prior to starting citalopram ensures that the clinical indicators purely reflect pre-treatment differences and are not conflated with early post-treatment symptom change, which may have more to do with treatment expectancies. See Supplemental Materials Section 1 for additional information about sample selection.

### Primary treatment response outcome

The aim was to classify participants as having had an adequate or inadequate response to treatment. We used the STAR*D treatment guidelines to guide the definition of treatment response. Specifically, these guidelines indicated that treatment should continue for at least 6 weeks, with sustained remission (defined as QIDS-C_16_ ≤ 5) for 2 weeks before moving a patient into follow-up. Patients who met this criterion were classified as having had an adequate response. At 9 weeks, if there was no response to treatment (defined as QIDS-C_16_ ≥ 9), the patient was moved to the next treatment level, and we classified these patients as having had an inadequate response.

If there was a partial response to treatment (defined as QIDS-C_16_ between 6 and 8), the clinician could either increase the dose or advance the patient to the next treatment level. If the patient still had a partial response but did not remit by 12 weeks (or 14 weeks if the clinician felt that remission could be achieved with an additional 2 weeks of treatment), then patients could either continue citalopram monotherapy (if they were satisfied with their improvement) or advance to the next treatment level (if they were not satisfied). Any patient who ultimately achieved QIDS-C_16_ ≤ 5 during this period was classified as having an adequate response, and any patient who regressed to having QIDS-C_16_ ≥ 9 was classified as having had an inadequate response. For those who remained in the ambiguous QIDS-C_16_ 6–8 range, we based the classification on whether or not they were satisfied with their treatment outcome (entered follow-up = adequate response) or dissatisfied with treatment (entered next treatment level = inadequate response). For additional rationale regarding our definition of treatment response, please see Supplemental Materials Section 2.

### Secondary outcomes

We identified two-related secondary outcomes: (1) *Achieving remission or satisfactory partial response, assuming medication tolerance.* This analysis excludes patients who exited Level 1 early because of intolerable side effects; otherwise, the outcome is defined the same as the primary outcome above. This definition of outcome is arguably more comparable to what has been used in previous studies and therefore may be the fairest test of the previously discovered SNPs^10^. (2) *The presence or absence of distressing side effects, regardless of treatment response.* In addition to withdrawing or level switching because of an adverse drug reaction, this outcome was defined as reporting, at the last clinic visit, at least one distressing side effect on the Patient Related Inventory of Side Effects (PRISE), or an overall intensity or burden of side effects that was at least “marked” on the Frequency, Intensity, and Burden of Side Effects Ratings (FIBSER).

### Candidate predictors

The goal was to identify as many potentially useful demographic, symptom, clinical, and genetic predictors of treatment response as possible. Total score and subscale scores if available (or individual items if not) were used as potential candidate predictors for self-report questionnaires. Prior to inclusion, all potential predictors were screened and eliminated for excessive (> 20%) missingness or near-constant values, defined as a single value observed for more than ~ 95% of cases (> 50 fewer than the total number of non-missing values). This criterion for near-constancy was chosen such that the data available for training the machine learner following data partitioning for nested cross-validation (~ 80% of original sample) and random subsampling (~ 50% of that subsample) would be expected to contain at least 20 examples (40% of 50) of the minority value(s) for any candidate predictor. This led to the exclusion of 1 variable for excessive missingness and several variables for near-constant values (more in the European-ancestry sample owing to its smaller sample size) for a final total of 164 predictor variables for the all-ancestry sample and 149 for the all-European sample. (The smaller sample size of the European-ancestry sample caused more variables to be excluded that had an insufficient number of contrasting examples. This obviously included the variables that identified race and ethnicity, as well as those that had insufficient examples of the following: (1) not experiencing anhedonia at enrollment, (2) comorbid panic or social phobia disorder, (3) visiting the ER for psychiatric reasons, and (4) several medication classes for those taking non-study meds.) Candidate predictors included age, race/ethnicity, depression symptoms measured with self-report (QIDS-SR) and interview (HRSD), MDD duration, antidepressant history, psychiatric comorbidity measured with the PDSQ, psychiatric history, family psychiatric history, physical illness, insurance status, disability, and mechanism of action of concomitant medication treatment. See Supplemental Table SM2 for a list of included variables and the proportion of missing values for each. Note that missing values were not imputed but rather passed “as is” to the machine learners.

### Genotyping procedures, imputation, variant reduction

Genetic data were obtained from the Center for Collaborative Genomic Studies on Mental Disorders (http://www.nimhgenetics.org). Genotyping for 500,453 markers on the 1948 subjects was conducted on two platforms. Nine hundred sixty-nine subjects were genotyped at Affymetrix on the Human Mapping 500 K Array Set. The remaining 979 samples were genotyped using the Affymetrix Genome-wide Human SNP Array 5.0. Validation using twelve samples genotyped on both the 500 K and 5.0 Arrays showed greater than 99% concordance in genotyped markers across the platforms^9,18^.

Quality control of the genotypic data was completed using PLINK v1.9^19^. SNPs were excluded if more than 2% of genotype data was missing. The threshold for minor allele frequency (MAF) was applied after phasing and imputation, as variant-level filtering has been shown to have a deleterious effect on imputation quality^20^. Moreover, we did not filter SNPs based on Hardy–Weinberg Equilibrium (HWE), as departures from HWE may be expected in a case-only sample. Samples were excluded on the basis of poor call rate, discordant self-reported and chromosomal sex, excessive autosomal heterozygozity, and relatedness. More detail about selecting the European subsample are provided in the Supplemental Materials Section 3.

Untyped variants were imputed on the Michigan Imputation Server (https://imputationserver.sph.umich.edu). Typed variants were phased with Eagle v2.4^21^ prior to imputation with Minimac4 v1.0.0^22^, using the 1000 Genomes Project Phase 3 v5^23^ as a reference panel. Following phasing and imputation, PLINK v2^19^ was used to apply further quality control to the imputed dosage data. SNPs with a MAF < 0.005 or imputation quality score < 0.90 were excluded from all statistical analyses. These high-quality SNPs were then pruned for linkage disequilibrium (LD) using PLINK v1.9, which identified a set of 371,868 approximately independent SNPs with a *R*^2^ threshold of 0.25, window size of 50 SNPs, and step size of 5 SNPs.

### A priori selection of SNPs

Prior work in an independent sample of 280 individuals identified 11 SNPs associated with depression response to escitalopram^10^. While only 4 of these 11 SNPs were available in the STAR*D dataset after implementing quality control procedures described above, we were able to recover up to 9 of the 11 SNPs by lowering the imputation quality score filter to < 0.30 (see Table 1). For the two SNPs that could not be recovered in STAR*D (rs151139256, rs2704022), we identified proxy SNPs that were in very strong LD with each missing variant (*r*^2^ > 0.98) using the European populations in 1000 Genomes Project Phase 3 v5 as the reference panel. Thus, in sum, we selected a priori 11 SNPs for the prediction models based on the prior work by Iniesta et al.^10^.

**Table 1:** A priori SNPs from^10^ shown to predict escitalopram response and the corresponding SNPs used in the STAR*D sample.

Iniesta SNPs	In STAR*D?	Best proxy	Proxy LD
rs1392611	Yes	N/A	N/A
rs10812099	Yes	N/A	N/A
rs1891943	Yes	N/A	N/A
rs151139256	No	rs62181046	1
rs11002001	Yes	N/A	N/A
rs62182022	Yes	N/A	N/A
rs28373080	Yes	N/A	N/A
rs7757702	Yes	N/A	N/A
rs76557116	Yes	N/A	N/A
rs9557363	Yes	N/A	N/A
rs2704022	No	rs1693558	0.9801

Proxy LD refers to *R*^2^ between the Iniesta SNP and the proxy SNP, as calculated by LDlink^47^ (https://ldlink.nci.nih.gov/). As the majority of the STAR*D sample is of European ancestry, we used the European populations in 1000 Genomes Project Phase 3 v5 as the reference panel.

### Selection of SNPs with elastic net logistic regression

As described above, candidate SNPs were first reduced to a set of 371,868 SNPs after removing highly correlated SNPs and SNPs with low variance. We then used an elastic net with predominantly lasso penalty (alpha = 0.99) to reduce the data to a smaller set of variants to combine with all other patient variables. Alpha was chosen to be near 1 on the assumption that only a small percentage of the 300,000 + SNPs would be relevant to prediction and that most of the coefficients are truly 0. This was done within a nested cross-validation procedure, so a potentially different set of SNPs was selected for each combination of 13 (14, minus 1 holdout) STAR*D centers (regional groupings of clinic sites) used as the training data.

### Learning algorithms and tuning parameters

To predict treatment outcome, we implemented a type of ensemble learning called stacking or super learning^24^. Stacking trains a second-level meta-learner to build an ensemble prediction based on the first-level predictions of a diverse set of base learners. More detail about the machine learning parameters are provided in the Supplemental Materials Section 4.

*Meta-learner*. A stacked ensemble of 100 Gradient Boosted Machines (GBMs) was trained, each with a randomly selected combination of tuning parameters, with predictions integrated by ridge regression.

### Prediction metrics and cross-validation

An important aspect of model performance is how well it performs on cases that it was not trained on. We used 14-fold cross-validation to estimate model performance, which reflects the mean predictive performance of the model in previously unseen data. In this case, we used 14-fold cross-validation because there were 14 geographic regional centers identified in the STAR*D trial dataset. Thus, the models were trained on 13 of the regional centers and then tested in the one hold-out center, essentially examining how well models trained on one set of study centers generalizes to a new study center, which may have more ecological validity for estimating how well the model will perform when implemented in a novel clinical setting. This process was repeated 14 times, with each regional center taking turns as the holdout sample, and then averaged across the repetitions.

### Differences between the current modeling approach and prior work with the STAR*D sample

There are a number of differences between the current modeling approach to prediction and prior work with the STAR*D sample that are important to highlight. First, while at least three studies have applied machine learning methods to the prediction of STAR*D outcomes, only one has predicted response to citalopram specifically^12,25,26^; the other two predicted treatment resistance more broadly^12,25,26^, defined as a failed response to citalopram (“Level 1”) plus one of several additional treatments (“Level 2”).

Another important distinction is that we excluded data that was collected after a patient started citalopram. This exclusion criterion has not been previously applied^12,25,26^. One study explicitly incorporated predictors collected 2 weeks after starting treatment; the other studies ostensibly aimed to identify pre-treatment predictors of treatment response but did not take into account the fact that patients were allowed to enroll in the STAR*D study after having already started citalopram and could therefore receive their “baseline” assessment as much as two weeks after starting treatment. Including clinical variables obtained after treatment initiation could be influenced by early symptom change, which has been shown to predict treatment response^27^, and may artificially inflate prediction accuracy.

We also used nested cross-validation based on regional center holdouts. It is important to understand that cross-validation has two uses in machine learning: to inform the optimization and selection of models and to provide an estimate of test error (out of sample generalization). Notably, if cross-validation error is used to optimize models, it no longer provides an unbiased estimate of test error. Nested cross-validation avoids this bias by nesting the cross-validation used for model optimization within a cross-validation used for estimating test error. Moreover, by basing the cross-validation folds on regional centers, we more closely mimic the expected test error when generalizing to an independent clinic.

Prior work aimed at predicting citalopram response used a feature selection step that appears to have been based on the entire STAR*D data set and reports classification metrics based on the same internal cross-validation used to optimize model parameters^12^. Consequently, while this study obtained an unbiased assessment of their model by testing it on an independent sample from a different clinical trial, the performance stats reported for the STAR*D sample are likely inflated, perhaps accounting in part for the large drop-off in performance between their cross-validation estimate and their independent estimate.

The studies predicting multi-treatment resistance did provide an unbiased estimate of site-to-site generalization by splitting the regional centers into independent samples for training and testing^25,26^. However, both these studies evaluated a single split whereas we evaluated the average of 14 such splits. In addition to assessing a different outcome on a more restricted sample and excluding predictors collected after the start of treatment, our validation strategy helped us to avoid reporting a fortuitous data partition; Table SM1 shows that while the average split performs worse than these prior models, 4/14 such splits would have performed almost as well or much better.

### Data analysis software

All analyses were implemented in R (version 4.0). Our code made extensive use of the *tidyverse*^28^ packages *dplyr*, *purrr,* and *tidyr* for general data extraction and transformation. The *SnpStats*^29^ and *glmnet*^30^ packages were used for processing and selecting SNP data, and *H2O*^31^ was used to implement the machine learning ensembles.

## Results

### Primary outcome: prediction of treatment response

The stacked ensemble model with pretreatment clinical predictors but no genetic variants had acceptable overall model performance, AUC = 0.659 (see Table 2). Notably, the stacked ensemble model with pre-treatment predictors plus the genetic variants selected a priori did not improve treatment outcome prediction beyond the stacked ensemble with only the clinical predictors—the 95% CIs were highly overlapping for both models (see model performance metrics in Table 2).

**Table 2:** Model performance for baseline features predicting treatment outcome.

Model	AUC (95% CI)	Accuracy %	Sensitivity	Specificity	R^2^
Clinical predictors	.659 (.629, .689)	.615	.623	.607	.057
Clinical predictors + a priori SNPs	.662 (.632, .692)	.628	.642	.613	.059
Clinical predictors + elastic net SNPs	.655 (.625, .685)	.609	.623	.594	.049

Clinical predictors model includes sociodemographic and pre-treatment symptom variables only. The model in the second row adds SNPs selected a priori based on work by^10^ to the clinical predictors model. The model in the third row adds the SNPs identified by the elastic net feature selection to the clinical predictors model. For threshold-dependent metrics (accuracy, sensitivity, specificity), a probability threshold of 0.5 was used for classification.

Similarly, the model that included the SNPs selected via elastic net also did not improve prediction beyond the clinical predictors model. Across the *k*-folds, the number of SNPs selected ranged from 0 to 71 with an average of 18 SNPs selected. Among the folds that did retain candidate predictor SNPs, a total of 227 unique SNPs were selected. However, none were observed in more than 4 of the folds (rs12371750 and rs1537728 were retained in 4 of the folds). In sum, there was very little consistency in which SNPs were retained during the selection process and the addition of these SNPs to the clinical predictors did not improve the prediction of treatment outcome. Model performance across the STAR*D geographic site locations is presented in the Supplemental Materials Section 5.

### Secondary outcome: prediction of distressing or intolerable side effects

We tested the same three models as before but with a different treatment outcome: the occurrence of distressing or intolerable side effects. (We did not include results from the a priori SNPs because they were not selected for the prediction of side effects.) The stacked ensemble model with pretreatment clinical predictors but no genetic variants had adequate performance, AUC = 0.618. Notably, the stacked ensemble model with pre-treatment predictors plus the genetic variants selected by elastic net did not improve prediction beyond the clinical predictors model (see Table 3).

**Table 3:** Model performance for baseline features predicting distressing or intolerable side effects treatment outcome.

Model	AUC (95% CI)	Accuracy	Sensitivity	Specificity	R^2^
Clinical predictors	.618 (.587, .649)	.577	.541	.611	.030
Clinical predictors + elastic net SNPs	.608 (.577, .639)	.583	.547	.617	.024

Clinical predictors model includes sociodemographic and pre-treatment symptom variables only. The model in the second row adds the SNPs identified by the elastic net feature selection to the clinical predictors model. For threshold-dependent metrics (accuracy, sensitivity, specificity), a probability threshold of 0.5 was used for classification.

### Secondary outcome: prediction of treatment response given no distressing or intolerable side effects

The stacked ensemble model with pretreatment clinical predictors but no genetic variants had acceptable model performance, AUC = 0.663 (see Table 4). Notably, the stacked ensemble model with pre-treatment predictors plus the genetic variants selected a priori did not improve treatment outcome prediction beyond the stacked ensemble with only the clinical predictors. Similarly, the model that included the SNPs selected via elastic net also did not improve prediction beyond the clinical predictors model. Thus, the addition of genetic variants to the clinical predictors did not improve the prediction of treatment outcome when distressing and intolerable side effects were removed from the definition of treatment outcome. Or, conversely, whether or not we included people with distressing and intolerable side effects as part of the operationalization of treatment outcome did not appear to strongly impact the results. The primary and secondary analyses were repeated in the subset of participants with European ancestry and are presented in Supplemental Materials Section 6. The conclusions were very similar to the main analyses.

**Table 4:** Model performance for baseline features when people that experienced distressing and intolerable side effects were removed from the treatment outcome.

Model	AUC (95% CI)	Accuracy	Sensitivity	Specificity	R^2^
Clinical predictors	.663 (.632, .694)	.617	.734	.470	.056
Clinical predictors + a priori SNPs	.661 (.629, .692)	.623	.740	.474	.055
Clinical predictors + elastic net SNPs	.656 (.625, .688)	.616	.723	.480	.048

Clinical predictors model includes sociodemographic and pre-treatment symptom variables only. The model in the second row adds SNPs selected a priori based on work by^10^ to the clinical predictors model.The model in the third row adds the SNPs identified by the elastic net feature selection to clinical predictors model. For threshold-dependent metrics (accuracy, sensitivity, specificity), a probability threshold of 0.5 was used for classification.

### Sensitivity analyses: SNP only prediction of outcomes

It may be that genetic variation was not a robust predictor of treatment outcome because it predicts variance in outcome that is redundant with the clinical variables. To address this possibility, the final analysis used the a priori SNPs and the SNPs selected by elastic net to predict the primary outcome in the absence of clinical predictors. The a priori SNPs only model did not perform well in the full sample (AUC = 0.481, 95% CI [0.454, 0.509]) or the European-ancestry sample AUC = 0.503, 95% CI [0.469, 0.537]. Similarly, the elastic net selected SNPs only model did not predict treatment response well in the full sample (AUC = 0.496, 95% CI [0.468, 0.524]) or the European-ancestry sample (AUC = 0.473, 95% CI [0.439, 0.507]).

## Conclusion

The current study builds upon prior work predicting treatment outcome in STAR*D by incorporating genetic variation and by using a stacked ensemble meta-learner algorithm to predict clinically relevant treatment outcomes in response to citalopram treatment. The main finding from this work is that common SNP variation, as implemented in the current study, did not improve prediction of response to citalopram in the STAR*D trial over and above the prediction provided by demographic and clinical variables.

We examined two methods for identifying potentially useful genetic variants—a priori selection of SNPs previously shown to predict treatment response to citalopram and an elastic-net approach to identify the most promising SNPs. Neither approach improved the prediction of treatment response—in fact, the inclusion of genetic variants tended to slightly impair model performance. The best performing stacked ensemble GBM that only used pre-treatment clinical and sociodemographic predictors had an AUC of 0.663 and model accuracy was 61.7%. This model performance is in line with prior work using a different machine learning approach, which reported an AUC of 0.700 and model accuracy of 64.6% for the prediction of treatment response (final score QIDS-SR_16_ < 6 at week 12 or week 14) in the larger STAR*D sample (not restricted to participants who provided DNA) using pre-treatment clinical predictors. Thus, there is quite a bit of room for prediction improvement; unfortunately, common SNP variation does not appear to offer any improvement. Indeed, the genetic variant only model (i.e., without any clinical predictors) did not outperform chance.

Given that citalopram treatment response appears to be modestly heritable, approximately 40% *in this sample*^4,5^, why did the inclusion of SNPs not improve the prediction of treatment response? It has been estimated that for complex traits, accurate prediction at the individual level is dependent on the heritability and prevalence of the complex trait. Simulations suggest that accurate prediction may require the genetic variants to capture a large proportion of the heritability in order to obtain an acceptable AUC^32^. Further, the effects of SNPs for this complex phenotype may be so small that they are difficult to estimate with high accuracy unless a very large discovery sample is used^33^. Although prior work identified SNPs that improved prediction of escitalopram response with much smaller samples (e.g., training set *N* = 280 and a validation set of *N* = 150 in^10^), that work did not report the results of a SNPs-only model or a clinical-predictors only model, so the additive value of the SNPs to their model is unknown. Further, they combined data from nine clinic sites and randomly partitioned it into a single train-test (65% / 35%) set; thus the problem of clinic-to-clinic generalization was not assessed. Results from the current study unfortunately suggest that their promising results may not generalize to other datasets.

Unfortunately, given the curse of dimensionality, there is likely no selection technique, including machine learning techniques such as those used in the current study, that avoids needing exceedingly large sample sizes to identify SNPs that provide a signal that will generalize out of sample. Indeed, there was high variability in SNPs selected by the elastic-net during the *k*-fold cross-validation procedure. Other work taking a candidate gene^6,7^, candidate system approach^5^, or polygenic risk scores^11^ have generally found similarly disappointing results for the prediction of antidepressant treatment response. Thus, genetic variation may be most useful for investigating the etiology of treatment response between groups of patients (e.g., responders vs non-responders) but may currently not be useful for deriving personalized predictions of treatment response^34^. Other areas of research, such as educational attainment, have arrived at similar conclusions^35^.

One alternative method for potentially improving individual-level prediction of baseline models is to add additional features beyond SNP variation. Future data-mining work may benefit from exploring more continuous measures of molecular variation, such as methylomic variation^36,37^ or hormonal profiles^38^, perhaps in combination with other neurobiological data^39–41^. However, psychosocial or behavioral data with strong psychometric properties^42^ should not be ignored, as they too could explain unique variance in antidepressant treatment response above and beyond neurobiological data^43^.

There are several limitations of this study that should be noted. First, without comparable data from an alternative intervention, we have no way of gauging the extent to which this model is predicting response to citalopram specifically versus response to interventions more generally. Notably, prior predictive modeling has shown some specificity to citalopram, as using the prognostic model developed for citalopram did not predict beyond chance response to a different antidepressant medication^12^. Second, in order to recapture most of the a priori SNPs^10^ and only use two high-LD proxy SNPs, we used a relatively low imputation threshold (< 30%). This low imputation threshold could partly account for why these SNPs did not improve treatment response prediction in this sample. In addition, Affymetrix arrays used in STAR*D tend to underperform compared with arrays produced by other manufacturers^44^ and imputation of non-genotyped SNPs may be suboptimal^45^. This could have contributed to the poor performance for the confirmatory SNPs selected based on prior work^10^. Finally, there are many published studies involving STAR*D participants, although none taking a similar approach to identify genetic variants and using them in the prediction of treatment outcome.

The STAR*D trial demonstrates what can be accomplished when large, multi-site trials are openly shared with other scientists for secondary analyses^46^. Open datasets of large pharmacologic and psychosocial interventions trials would greatly facilitate further development of treatment outcome algorithms for a variety of treatments. This could lead to the development of a database of algorithms that clinicians and patients could use to help make clinical treatment decisions. Making such data widely available could therefore promote a more efficient mental health care system by helping clinicians optimize treatment delivery to specific patients with the goal of receiving the treatment with the best likelihood of a successful response as quickly as possible. Currently, the usefulness of SNP variation for tailoring treatment to patients appears uncertain, as reliable SNP predictors of antidepressant treatment response have yet to be identified.

## Supplementary Information

Supplementary Information.
