# Supplementary material for: Inclusion of genetic variants in an ensemble of gradient boosting decision trees does not improve the prediction of citalopram treatment response
Source: Sci Rep. 2021 Feb 12;11:3780. doi: 10.1038/s41598-021-83338-2 (PMC7881144; doi:10.1038/s41598-021-83338-2)
Supplement: Supplementary file 1 — Supplementary Information. [file 41598_2021_83338_MOESM1_ESM.docx]

Supplementary material for:

Inclusion of genetic variants in an ensemble of gradient boosting decision trees does not improve the prediction of citalopram treatment response

^1^Jason Shumake, ^1^Travis T. Mallard, ^2^John E. McGeary, ^1^Christopher G. Beevers

^1^University of Texas at Austin, Austin, TX, USA

^2^Providence Veterans Affairs Hospital and Brown University School of Medicine, Providence, RI, USA

**Supplemental Materials**

1. Rationale for sample selection

2. Additional rationale for the operationalization of treatment response

3. Methods for determining European subsample

4. Learning algorithms supplemental information

5. Model performance by geographic region

6. Sensitivity Analyses: European Ancestry Sample Only

7. Complete List of Non-genomic Predictors Used in Models.

References

All analytic code, results, and reports (RMarkdown in html format) for this project can be found here: https://dataverse.tdl.org/dataverse/snp_pred. STAR*D data is publically available from the NIMH Data Archive: https://nda.nih.gov/.

## 1. Rationale for sample selection

We used the full sample in our primary analyses (rather than limiting our analyses to European ancestry) because the machine learning approach we used is capable of discovering conditional relationships, such as utilizing different sets of SNPs (or ignoring SNPs altogether) conditional on a person’s race or ethnicity. This would obviate one of the primary arguments against multiethnic samples, which is that a SNP could be predictive of treatment outcome in one population but not another (creating a confound between the allele frequency and ethnic background). For example, a decision tree in a machine-learner ensemble could handle this scenario by creating separate branches for white and non-white patients, and then producing additional branches for the white branch that modify its prediction based on SNPs that are only predictive for the white population. We therefore opted to maximize our sample size by including all valid patient data. However, it is also possible that the numbers of racial-ethnic minorities in this sample were not large enough to reliably discover such conditional rules, in which case their inclusion could introduce more noise than signal and thereby interfere with the discovery of predictive SNPs for the white European majority. Thus, to address this possibility and to facilitate comparison to other studies which are limited to European ancestry, we also repeated all models restricted to this subsample.

## 2. Additional rationale for the operationalization of treatment response

This approach to defining the primary outcome differs from previous studies which have adhered to an inflexible score cutoff for classification (e.g., QIDS <= 5 vs QIDS > 5), which ignores the clinical reality that a QIDS score of 5 is not meaningfully different from a QIDS score of 6; we believe that classifying based on the actual treatment decision is a more valid method of dichotomizing cases that are “on the fence” of being remitted vs. not remitted.

Further, previous studies have tended to exclude cases who exited the citalopram-monotherapy level early, on the grounds that these patients did not receive an adequate trial to determine treatment efficacy. However, many of the patients who exited early did so because of emergent or worsening symptoms and/or suicide attempts requiring non-protocol treatment or hospitalization, or because of citalopram-related serious adverse events or intolerable side effects. When such conditions were documented at level exit, we also classified these cases as having had an inadequate response; prescribing citalopram was not a good treatment decision for these patients. For anyone else who dropped out early or who was labeled as protocol non-adherent in the absence of intolerable side effects, the outcome was considered unknown.

Our primary outcome used a clinically-relevant definition of treatment outcome, including stopping treatment due to side effects. However, prior work predicting antidepressant treatment response did not include side effect response in the definition of treatment response. Thus, we performed a follow-up analysis that excludes patients who exited Level 1 treatment early because of intolerable side effects (see Secondary Outcomes).

## 3. Methods for determining European subsample

Principal components of ancestry were estimated with flashPCA2 [1] using the 1000 Genomes Project Phase 3 v5 as a reference panel. While we used the full multi-ethnic sample in our primary analyses, we also performed sensitivity analyses in participants of European ancestry to evaluate potential population stratification. To identify the relatively homogenous European subsample, we first selected participants who identified as “Not Hispanic or Latino” and “White” in STAR*D with usable genetic data. We then computed the mean of the first two PCs of ancestry for the European reference samples before calculating the maximum Euclidean distance from the center point. This parameter was then multiplied by a conservative scalar and used to demarcate the boundaries of the European ancestry cluster, excluding individuals who were designated as outliers (see Figure SM1 for more detail) . Collectively, these quality control procedures yielded a full sample of 1,782 and a European-only sample of 1,170.


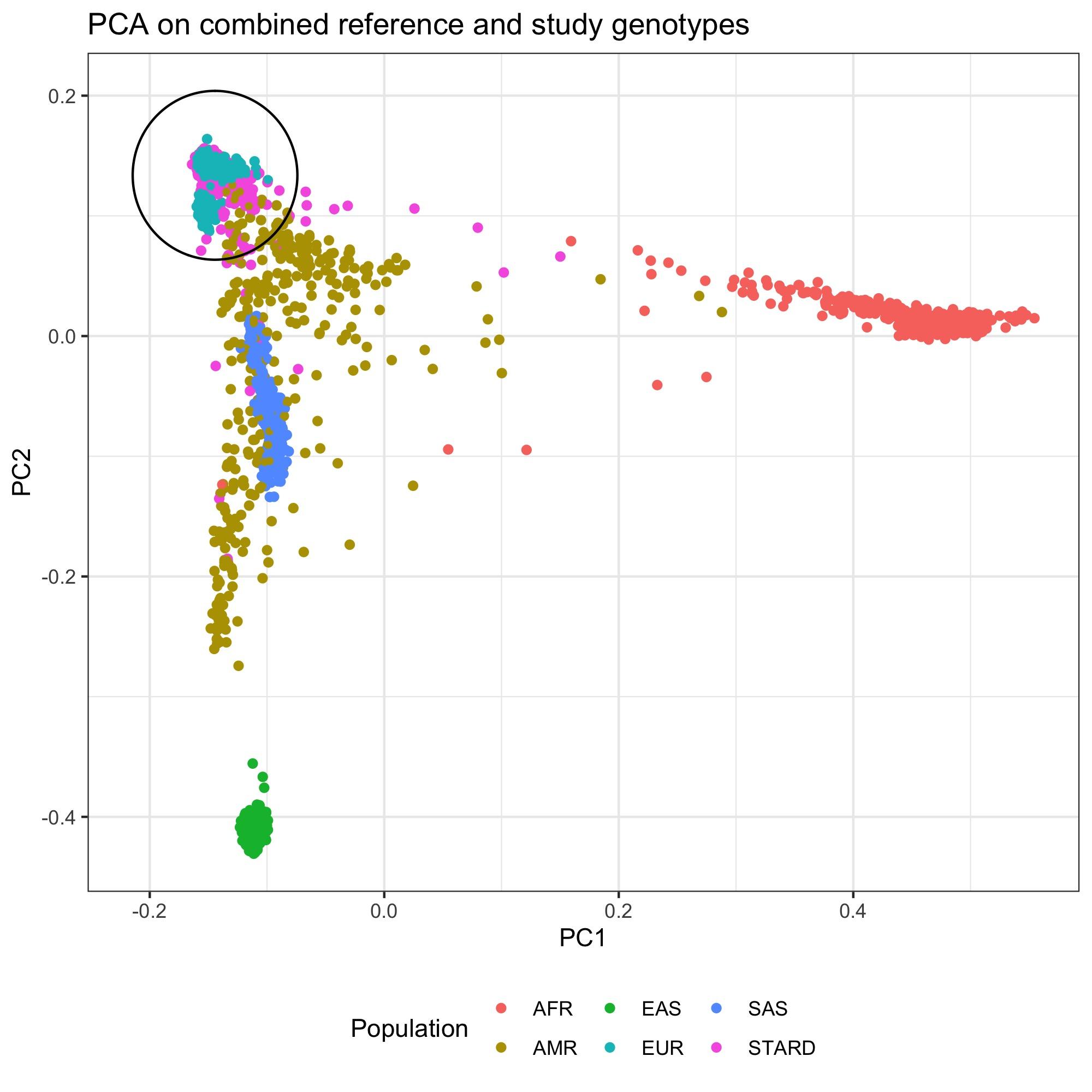


Figure SM1. Principal component analysis on combined reference and study genotypes. The black circle in that figure demarcates the boundary of non-Hispanic European ancestry (using 1KG as the reference data). STAR*D participants (in pink) who are outside the boundary were identified as ancestral outliers, and subsequently removed from analyses focusing on the homogenous subsample of non-Hispanic European participants in the sensitivity analyses.

## 4. Learning algorithms supplemental information

*Base models*. The base learners were trained using the *h2o.gbm* algorithm. We chose to stack a grid search from this same base learner because of its implicit handling of missing data and indifference to diversity of variable types and scales, allowing us to avoid preprocessing imputation and scaling procedures which would be necessary for many other methods (e.g., regularized linear regression). In addition, GBMs have a very large hyperparameter space, which can lead to diverse predictions between differentially tuned models, even within the same base algorithm.

The typical approach is to stack models generated from different learning algorithms (e.g., ensembling together a GLM, random forest, and a GBM). An alternative approach is to stack multiple models generated from the same base learner but tuned under a diverse set of parameters[2]. The traditional approach is to perform a grid search over many possible combinations of tuning parameters and then use cross-validation to select the single best performing model. However, certain tuning parameters, which might be suboptimal in terms of overall prediction error, may nonetheless identify unique patterns within the data that are missed by the winning model. Rather than discard all these models, we can capitalize on their unique benefits by combining them into a meta-model. This goes against the common intuition that removing models that are weak performers by themselves should increase the performance of an ensemble; in fact, removing weak learners is often detrimental to ensemble performance.

*Varied parameters*. A random grid search explored the following space of parameters: (1) Whether or not to balance the classes via over/under-sampling, and if oversampling is used (observations from the minority class are randomly duplicated), the factor by which the size of the training data is allowed to grow (1-10 in integer increments). For example, a factor of 1 means that for every observation from the minority class that is duplicated, an observation from the majority class must be deleted. 2) Interaction depth: 1-9 in integer increments. 3) Sampling rate for rows (observations): 0.2 - 1 in 0.05 increments. 4) Sampling rate for columns (predictors) per tree: 0.2 - 1 in 0.05 increments. 5) Sampling rate for columns (predictors) per split: 0.2 - 1 in 0.05 increments. Note that this refers to the fraction of available predictors per tree, so the effective sampling rate for columns is multiplicative across the per-tree and per-split rates. For example, a per-tree sampling rate of 0.5 and a per-split sampling rate of 0.5 means that the split will be based on 25% of the predictors. 6) Sampling rate change for columns as a function of split depth: 0.9 - 1.1 in 0.01 increments. This allows for the column sampling rate (#5) to become more or less random as branches are added to a given tree. 7) Minimum number of observations in a terminal node. The smaller this number is, the more individualized (but less generalizable) the predictions will be. The search space for this parameter was defined by the following logarithmic sequence: 1, 2, 4, 8, 16, 32, 64, 128, 256. 8) Minimum fractional improvement in error required for partitioning to continue: 0, 1×10^8^, 1×10^6^, 1×10^4^. For example, when this parameter is set to 0, tree splitting will continue until the minimum terminal node size (#7) is reached; otherwise, splitting might be stopped before this node size limit is reached, if the relative error improvement would be less than the given fraction. 9) Histogram type: “UniformAdaptive”, “Random”, “QuantilesGlobal”, or “RoundRobin”. This controls how numeric data are binned prior to searching for optimal split points. For each stacked-ensemble model, 100 GBMs were trained, each using a different random combination of values for all the above parameters.

*Fixed parameters*. In addition to these variable parameters, the following parameters were fixed for all models: 1) Each GBM was allowed to grow to a maximum of 10,000 trees, but an early stopping rule was used that terminated model expansion when the simple moving average of log loss improved by less than 0.1% over the last 50 trees. 2) Simulated annealing was used to dynamically adjust the learning rate of each GBM. The learning rate was initialized at 0.05 (a relatively fast learning rate) and then reduced by a factor of 0.99 after every tree. This results, for example, in a learning rate of 0.018 after 100 trees and 0.007 (a relatively slow learning rate) after 200 trees. Slower learning rates generally result in better predictions but require more trees to be fit; annealing is a way to optimize the trade-off between computation speed and prediction accuracy (large increase in speed without large loss of accuracy). Especially since these GBMs are not intended to provide a final prediction but rather to provide inputs to a subsequent meta-learner, the individual GBMs do not need to be maximally generalizable; rather, the objective is to sample a large and diverse space of tuning parameters, and then let the meta-learner optimize generalizability across the stacked ensemble of GBMs. For any other model parameters not specified above, the h2o.gbm default values were used.

## 5. Model performance by geographic region

It may also be informative to examine model performance for the primary outcome based on geographic region. This analysis examines how well each prediction model that is trained and averaged across many geographic regions generalizes to a new, unseen region. That is, how well does data from all the other regions generalize to a new region? Notably, there was some variation in model performance by geographic region (see Table 3). In most regions the clinical-predictors-only model outperformed the models that included SNPs identified a priori or by elastic net, with a couple of exceptions. For example, if one were to single out Regional Centers 4 and 12 as a test set, one might conclude that the addition of genes selected by the elastic net led to a marginal improvement in prediction. But this belies the fact that, on average, the addition of these genes to the model leads to marginally worse predictions. Despite these two exceptions, in general, the prediction model had good generalization across geographic regions (AUCs for the clinical predictors only model ranged from .569 to .772).

| *Table SM1*. Overall model performance (AUC) for each model by geographic region. | | | | | | |
| --- | --- | --- | --- | --- | --- | --- |
| **Regional Center** | **Region** | **N** | **Response Rate** | **Clinical predictors** | **a priori SNPs** | **elastic net SNPs** |
| 1 | West | 135 | 40% | 0.71 | 0.69 | 0.70 |
| 2 | South | 86 | 40% | 0.63 | 0.65 | 0.62 |
| 3 | Midwest | 83 | 64% | 0.58 | 0.57 | 0.60 |
| 4 | Midwest | 89 | 55% | 0.66 | 0.67 | 0.70 |
| 5 | South | 60 | 55% | 0.60 | 0.63 | 0.61 |
| 6 | South | 68 | 50% | 0.57 | 0.58 | 0.58 |
| 7 | South | 109 | 51% | 0.70 | 0.70 | 0.71 |
| 8 | West | 142 | 47% | 0.69 | 0.70 | 0.65 |
| 10 | South | 140 | 46% | 0.73 | 0.73 | 0.73 |
| 11 | Midwest | 36 | 69% | 0.68 | 0.69 | 0.67 |
| 12 | Northeast | 78 | 49% | 0.56 | 0.57 | 0.60 |
| 13 | South | 66 | 68% | 0.77 | 0.78 | 0.77 |
| 14 | Northeast | 92 | 47% | 0.58 | 0.60 | 0.55 |
| 15 | Northeast | 73 | 59% | 0.64 | 0.65 | 0.66 |
| *Note*: Regional center grouping was determined by investigators in the STAR*D trial (there is no regional center #9). *N* reflects the number of participants from the genetics subset included in each geographic region. The clinical predictors model only includes sociodemographic and pre-treatment symptom variables. The “elastic net SNPs model” adds the SNPs identified by the elastic net feature selection to the clinical predictors model. The “a priori SNPs” model adds SNPs associated with escitalopram treatment response identified by prior work by Iniesta and colleagues [3] to the clinical predictors model. | | | | | | |

## 6. Sensitivity Analyses: European Ancestry Sample Only

We repeated the primary and secondary analyses in the subset of participants with European ancestry. For the primary outcome, all models appear to perform marginally better than what was observed in the full sample; however, the pattern within this sample was the same. The ROC curves when using training data sets that included selected SNPs, either based on previous literature (AUC = 0.671, 95% CI [0.634, 0.708]) or selected using an elastic net regression (AUC = 0.666, 95% CI [0.629, 0.702]) was not superior to training data that included only clinical predictors (AUC = 0.674, 95% CI [0.637, 0.71]).

When examined by geographic region, performance at some sites appeared to perform quite well when the models excluded non-European ancestries (e.g., Regional Center 1, AUC for the clinical predictors only was .791 in the European ancestry sample versus .705 in the full sample). Whether this has to do with differences in ethnic compositions between regions or reflects random error is somewhat difficult to determine given the relatively small sample sizes for some of the study sites. Nevertheless, the overall pattern is clear: the inclusion of genetic variants did not improve model performance.

For the secondary outcome of positive treatment outcome given no intolerable side effects, the ROC curves when using training data sets that included SNPs, selected either a priori (AUC = 0.668, 95% CI [0.629, 0.707]) or using an elastic net regression (AUC = 0.663, 95% CI [0.624, 0.703]) was not superior to training data that included only clinical predictors (AUC = 0.67, 95% CI [0.631, 0.709]).

Notably, models predicting side effects perform worse when limited to European ancestry. Once again there is no apparent value of adding genetic predictors. The ROC curves when using training data sets that selected SNPs using an elastic net regression (AUC = 0.558, 95% CI [0.519, 0.597]) was not superior to training data that included only clinical predictors (AUC = 0.571, 95% CI [0.532, 0.61]). In sum, these sensitivity analyses were generally consistent with the full sample and indicate that the addition of genetic variants does not improve prediction of treatment outcome or experience of intolerable side effects.

## 7. Complete List of Non-genomic Predictors Used in Models.

Table SM2. List of all predictors grouped by category with missing-value incidence for the full and racially homogeneous samples. The “—“ symbol indicates variables that were dropped due to an insufficient number of minority-class examples in the smaller sample.

| Predictor | Fraction missing (all ancestries) | Fraction missing (European ancestry) |
| --- | --- | --- |
| Comorbid Diagnoses | | |
| panic disorder | 0.00 | — |
| social phobia | 0.00 | — |
| posttraumatic stress disorder | 0.00 | 0.00 |
| generalized anxiety disorder | 0.00 | 0.00 |
| Demographics | | |
| home ownership | 0.15 | 0.13 |
| income | 0.11 | 0.10 |
| source of income | 0.11 | 0.11 |
| race | 0.08 | — |
| health insurance status | 0.02 | 0.02 |
| education (years of) | 0.00 | 0.00 |
| on medical leave | 0.00 | 0.00 |
| years at current residence | 0.00 | 0.00 |
| household size, number relatives | 0.00 | 0.00 |
| household size, number friends | 0.00 | 0.00 |
| household size, total number | 0.00 | 0.00 |
| performs volunteer work | 0.00 | 0.00 |
| ethnicity | 0.00 | — |
| age | 0.00 | 0.00 |
| gender | 0.00 | 0.00 |
| type of residence | 0.00 | 0.00 |
| marital status | 0.00 | 0.00 |
| education (highest degree) | 0.00 | 0.00 |
| student status | 0.00 | 0.00 |
| employment status | 0.00 | 0.00 |
| Depression Diagnosis | | |
| depressed mood | 0.00 | 0.00 |
| loss of interest/pleasure | 0.00 | — |
| weight loss or gain | 0.00 | 0.00 |
| insomnia or hypersomnia | 0.00 | 0.00 |
| psychomotor agitation or retardation | 0.00 | 0.00 |
| fatigue | 0.00 | 0.00 |
| feeling worthless or inappropriate guilt | 0.00 | 0.00 |
| decreased concentration | 0.00 | 0.00 |
| thoughts of death/suicide | 0.00 | 0.00 |
| Family History | | |
| bipolar disorder | 0.01 | 0.01 |
| alcohol abuse or dependence | 0.01 | 0.01 |
| unipolar depression | 0.01 | 0.01 |
| drug abuse or dependence | 0.01 | 0.01 |
| Hamilton Rating Scale for Depression (HRSD) | | |
| appetite | 0.00 | 0.00 |
| weight | 0.00 | 0.00 |
| early insomnia | 0.00 | 0.00 |
| middle insomnia | 0.00 | 0.00 |
| late insomnia | 0.00 | 0.00 |
| depressed mood | 0.00 | 0.00 |
| anxiety | 0.00 | 0.00 |
| insight | 0.00 | 0.00 |
| somatization | 0.00 | 0.00 |
| hypochondriasis | 0.00 | 0.00 |
| guilt | 0.00 | 0.00 |
| suicidality | 0.00 | 0.00 |
| loss of interest | 0.00 | 0.00 |
| fatigue | 0.00 | 0.00 |
| slowing | 0.00 | 0.00 |
| agitation | 0.00 | 0.00 |
| libido | 0.00 | 0.00 |
| total depression | 0.00 | 0.00 |
| Medical History | | |
| recent healthcare visits | 0.06 | 0.05 |
| emergency room visits | 0.06 | 0.05 |
| number of current medications | 0.01 | 0.00 |
| cardiac disease | 0.00 | 0.00 |
| hypertension | 0.00 | 0.00 |
| vascular disease | 0.00 | 0.00 |
| problem with eyes, ears, nose, or throat | 0.00 | 0.00 |
| upper gastrointestinal disease | 0.00 | 0.00 |
| lower gastrointestinal disease | 0.00 | 0.00 |
| renal disease | 0.00 | 0.00 |
| genitourinary disease | 0.00 | 0.00 |
| musculoskeletal disorder | 0.00 | 0.00 |
| neurological disease | 0.00 | 0.00 |
| psychiatric illness | 0.00 | 0.00 |
| respiratory illness | 0.00 | 0.00 |
| hepatic or pancreatic disease | 0.00 | 0.00 |
| endocrine disorder | 0.00 | 0.00 |
| cumulative illness rating | 0.00 | 0.00 |
| Medications Taken During Study | | |
| acetaminophen | 0.01 | 0.00 |
| adrenergic beta2-agonist | 0.01 | 0.00 |
| benzodiazepine | 0.01 | 0.00 |
| beta-adrenergic blocker | 0.01 | 0.00 |
| biguanide | 0.01 | — |
| calcium channel antagonists | 0.01 | — |
| corticosteroid | 0.01 | 0.00 |
| cyclooxygenase inhibitors | 0.01 | 0.00 |
| cytochrome P450 2C19 inhibitors | 0.01 | — |
| dihydropyridine calcium channel blocker | 0.01 | — |
| enzyme inhibitors | 0.01 | 0.00 |
| estrogen receptor agonist | 0.01 | 0.00 |
| full opioid agonists | 0.01 | — |
| GABA A agonists | 0.01 | 0.00 |
| histamine H2 receptor antagonist | 0.01 | — |
| histamine-1 receptor antagonist | 0.01 | 0.00 |
| HMG-CoA reductase inhibitor | 0.01 | 0.00 |
| any homeopathic remedy | 0.01 | 0.00 |
| insulin receptor agonist | 0.01 | — |
| l-thyroxine | 0.01 | — |
| opioid agonist | 0.01 | 0.00 |
| platelet aggregation inhibitor | 0.01 | 0.00 |
| progestin | 0.01 | 0.00 |
| proton pump inhibitor | 0.01 | 0.00 |
| serotonin reuptake inhibitor (additional) | 0.01 | 0.00 |
| sulfonylurea | 0.01 | — |
| thiazide diuretic | 0.01 | 0.00 |
| Miscellaneous | | |
| treatment expectancy | 0.00 | 0.00 |
| family impact | 0.00 | 0.00 |
| undocumented citalopram start date | 0.00 | 0.00 |
| days elapsed between first assessment and treatment start | 0.00 | 0.00 |
| menopausal status | 0.00 | 0.00 |
| Psychiatric Diagnostic Screening Questionnaire (PDSQ) | | |
| drug abuse or dependence | 0.02 | 0.01 |
| somatoform disorder | 0.01 | 0.01 |
| hypochondriasis | 0.01 | 0.01 |
| generalized anxiety disorder | 0.01 | 0.01 |
| major depressive disorder | 0.01 | 0.01 |
| dysthymic disorder | 0.01 | 0.01 |
| posttraumatic stress disorder | 0.01 | 0.01 |
| eating disorder | 0.01 | 0.01 |
| obsessive-compulsive disorder | 0.01 | 0.01 |
| panic disorder | 0.01 | 0.01 |
| mania | 0.01 | 0.01 |
| psychosis | 0.01 | 0.01 |
| agoraphobia | 0.01 | 0.01 |
| social phobia | 0.01 | 0.01 |
| alcohol abuse or dependence | 0.01 | 0.01 |
| total psychopathology | 0.01 | 0.01 |
| Psychiatric History | | |
| number MDD episodes | 0.14 | 0.14 |
| switching from another antidepressant | 0.07 | 0.08 |
| outpatient visits, not related to depression | 0.06 | 0.05 |
| emergency room visits, psychiatric | 0.06 | — |
| outpatient visits, related to depression | 0.06 | 0.05 |
| age of MDD onset | 0.01 | 0.01 |
| duration of current MDD episode | 0.00 | 0.00 |
| taken any psychiatric medication before | 0.00 | 0.00 |
| number of SSRIs already tried | 0.00 | 0.00 |
| SSRI response history | 0.00 | 0.00 |
| number of antidepressants tried before | 0.00 | 0.00 |
| antidepressant response history | 0.00 | 0.00 |
| has attempted suicide | 0.00 | 0.00 |
| Quick Inventory of Depression Symptoms (QIDS) | | |
| early insomnia (self-report) | 0.00 | 0.00 |
| middle insomnia (self-report) | 0.00 | 0.00 |
| late insomnia (self-report) | 0.00 | 0.00 |
| hypersomnia (self-report) | 0.00 | 0.00 |
| sadness (self-report) | 0.00 | 0.00 |
| indecision (self-report) | 0.00 | 0.00 |
| self-view (self-report) | 0.00 | 0.00 |
| suicidality (self-report) | 0.00 | 0.00 |
| interests (self-report) | 0.00 | 0.00 |
| energy (self-report) | 0.00 | 0.00 |
| slowing (self-report) | 0.00 | 0.00 |
| agitation (self-report) | 0.00 | 0.00 |
| appetite change (self-report) | 0.00 | 0.00 |
| weight change (self-report) | 0.00 | 0.00 |
| total depression (self-report) | 0.00 | 0.00 |
| hypersomnia (clinician) | 0.00 | 0.00 |
| early insomnia (clinician) | 0.00 | 0.00 |
| middle insomnia (clinician) | 0.00 | 0.00 |
| late insomnia (clinician) | 0.00 | 0.00 |
| sadness (clinician) | 0.00 | 0.00 |
| indecision (clinician) | 0.00 | 0.00 |
| self-view (clinician) | 0.00 | 0.00 |
| suicidality (clinician) | 0.00 | 0.00 |
| loss of interest (clinician) | 0.00 | 0.00 |
| energy (clinician) | 0.00 | 0.00 |
| slowing (clinician) | 0.00 | 0.00 |
| agitation (clinician) | 0.00 | 0.00 |
| appetite change (clinician) | 0.00 | 0.00 |
| weight change (clinician) | 0.00 | 0.00 |
| total depression (clinician) | 0.00 | 0.00 |

## References

1. Abraham G, Qiu Y, Inouye M. FlashPCA2: principal component analysis of Biobank-scale genotype datasets. Bioinformatics. 2017;33:2776–2778.

2. Boehmke B, Greenwell B. Hands-On Machine Learning with R. 2019.

3. Iniesta R, Hodgson K, Stahl D, Malki K, Maier W, Rietschel M, et al. Antidepressant drug-specific prediction of depression treatment outcomes from genetic and clinical variables. Sci Rep. 2018;8:5530.
